# Simulation Analysis of Air Flow and Turbulence Statistics in a Rib Grit Roughened Duct

**DOI:** 10.1155/2014/791513

**Published:** 2014-06-25

**Authors:** I. I. Vogiatzis, A. C. Denizopoulou, G. K. Ntinas, V. P. Fragos

**Affiliations:** ^1^Department of Hydraulics, Soil Sciences and Agricultural Engineering, Faculty of Agriculture, Aristotle University of Thessaloniki, 57001 Thessaloniki, Greece; ^2^Department of Electrical and Computer Engineering, Democritus University of Thrace, 67100 Xanthi, Greece

## Abstract

The implementation of variable artificial roughness patterns on a surface is an effective technique to enhance the rate of heat transfer to fluid flow in the ducts of solar air heaters. Different geometries of roughness elements investigated have demonstrated the pivotal role that vortices and associated turbulence have on the heat transfer characteristics of solar air heater ducts by increasing the convective heat transfer coefficient. In this paper we investigate the two-dimensional, turbulent, unsteady flow around rectangular ribs of variable aspect ratios by directly solving the transient Navier-Stokes and continuity equations using the finite elements method. Flow characteristics and several aspects of turbulent flow are presented and discussed including velocity components and statistics of turbulence. The results reveal the impact that different rib lengths have on the computed mean quantities and turbulence statistics of the flow. The computed turbulence parameters show a clear tendency to diminish downstream with increasing rib length. Furthermore, the applied numerical method is capable of capturing small-scale flow structures resulting from the direct solution of Navier-Stokes and continuity equations.

## 1. Introduction

Turbulent flow is a complex phenomenon even for simple geometries like square obstacles mounted on a surface which finds application in many physical and engineering problems. Such flows occur across a wide range of applications from aerodynamics, heat exchange systems to solar air heaters. For the flow investigation, researchers usually conduct experimental and numerical analyses in both two and three dimensions. The analyses focus on flow either around the sides of a surface-mounted obstacle, which is characterized as three-dimensional, or around a surface-mounted rib where the flow is then characterized as two-dimensional provided that the rib extends to the walls of the duct/simulation domain in the spanwise direction.

Because of their diverse and fundamental significance, numerous investigations both experimental as well as numerical have been conducted to particularly examine turbulent flow around two-dimensional surface-mounted ribs. The geometry that is commonly used when studying two-dimensional turbulent flows consists of square ribs. Such two-dimensional obstacles have been employed from a number of researchers to deduce conclusions about the different aspects of the respective turbulent flows. For example, Acharya et al. [1], Hwang et al. [2], and Panigrahi and Acharya [3] examined different aspects of flow movement and how it is modulated when using a surface-mounted square rib. More recently, Panigrahi et al. [4] experimentally investigated the reattaching shear layer development behind a surface-mounted square rib. The same geometry has been also used by Liu et al. [5] and Shah and Tachie [6] to investigate the shedding and flapping of vortical flows and the dynamics of separated and reattached flows, respectively, caused by a surface-mounted rib. An extension to the above experimental studies was the investigation of turbulent flow modulation when the flow passes over a series of square rib elements to draw useful conclusions concerning turbulence characteristics [7] and the wall roughness influence on flow configuration and heat transfer [8–10].

From the above it is evident that the study of two-dimensional, turbulent, separated, and reattached flow around ribs possesses a big portion of the researchers' interest due to the fact that such flow is frequently encountered in many technological and engineering applications. In particular, turbulent flow around ribs has practical importance when studying how fluid-related devices, such as gas turbines, turbo machines, and heat exchange systems, behave under certain flow conditions. Additionally, ribs serve as artificial roughness elements in a fluid flowing channel to improve the convective heat transfer by creating turbulence in the flow. This technique is of particular interest in renewable energy-related applications, especially when investigating the surface roughness effect and heat transfer in solar air heater devices where roughness element geometry is the most important parameter influencing the turbulent flow characteristics [11–13].

However, the experimental investigation of such phenomenon in order to find the optimum roughness pattern among different geometries is often a time consuming and tiresome process [14]. In this regard, the scope of the present study is to assess the flow behavior over surface-mounted ribs of varying lengths by using a dynamic numerical approach based on computational aspects and technique which, in many aspects, saves time and reduces cost. More specifically, the current work is investigating how different rib lengths affect the two-dimensional turbulent flow and turbulence characteristics such as turbulent intensity and turbulent kinetic energy for the case of *Re* = 1000. Moreover, the statistical processing of the simulation results depicts the impact that different rib lengths have on the separation and reattachment points of the flow, which directly affect the rate of heat transfer ([15], and references therein).

## 2. Simulation Setup Aspects

The results presented here are derived by executing a modified version of a noncommercial Fortran code initially developed by Fragos et al. [17], which employs the standard Galerkin finite elements method [18, 19] to solve the governing equations along with the appropriate initial and boundary conditions. The code has been experimentally verified by investigating the airflow patterns around obstacles with arched and pitched roofs [20]. The fluid is considered to have constant viscosity *ν* and density *ρ*. A uniform stream flow with velocity *U*_0_ is used as boundary condition at the entrance of the computational domain. The fluid moves uniformly through the duct until it hits the surface-mounted rib of height *h* with a 90° angle of attack. No-slip boundary conditions are imposed along the walls of the duct and the rib where the fluid is decelerated. The outlet boundary condition is a free boundary condition which lets the fluid exit the computational domain freely without any distortion.

The two-dimensional turbulent flow is assumed to have a constant temperature and the fluid to be incompressible. Under these assumptions the flow behavior can be accurately estimated by directly solving the dimensionless Navier-Stokes and continuity equations which represent conservation of momentum and mass, respectively.

Consider

**Figure 1:**
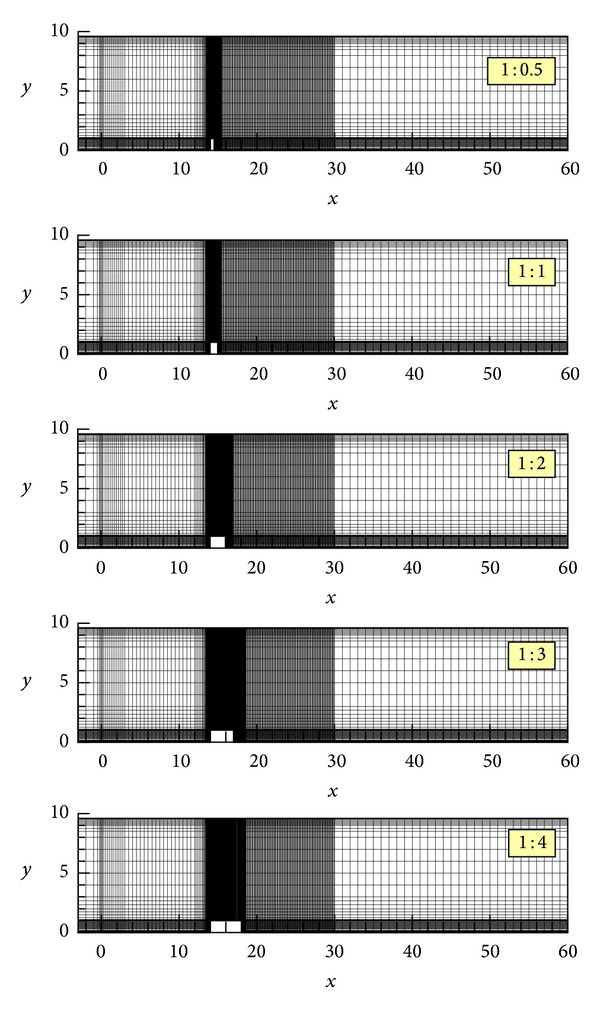
Computational meshes for the five rib cases.

(1) ∂Ui∂t+Uj∂Ui∂ωj=−∂P∂ωi+1Re∂2Ui∂ωj∂ωj,∂Ui∂ωi=0,

where tensor notation has been used and *i*, *j* are dummy indices which go from 1 to 2 and stand for *x* and *y* coordinates, respectively. *U*_*i*_ are the instantaneous components of the velocity vector, *P* is the pressure, and *Re* is the Reynolds number. *T*_*n*_ is the total time, which in the simulation runs is 150 dimensionless time units. The above equations have been rendered dimensionless by choosing *U*_0_ and *h* as the characteristic velocity and length, respectively. *Re* = *U*_0_ *h*/*ν* is the Reynolds number, where *ν* is the kinematic viscosity of the fluid and *h* is the rib height. Additionally, the pressure *P* and time *t* have been normalized by using the terms *ρU*_0_^2^ and *t*_*r*_, where *t*_*r*_ is defined by the ratio *h*/*U*_0_. The values of the input parameters yield a Reynolds number of 1000. However, it should be borne in mind that the flow is fully turbulent when using the duct height *H* for the calculation of the Reynolds number. The computational meshes used in the present study for the variable rib lengths are shown in Figure 1. They consist of rectangular finite elements of different sizes with nine nodes in each of them. The unknown velocities *U*_*i*_ and the pressure *P* of the governing equations (1) are expanded in terms of Galerkin basis functions as

(2) U1=∑k=19U1kϕk,  U2=∑k=19U2kϕk,P=∑k=14Pkψk,

where *ϕ* ^*k*^, *ψ* ^*k*^ are the quadratic and linear basis functions in each element, respectively.

The governing equations, weighted integrally with the basis functions, result in the following continuity, *R*_*C*_^*k*^, and momentum, *R*_*M*_^*k*^, residuals,

(3) RCk=∫V∇·UψkdV,

(4) RMk=∫V[∂U∂t+U·∇U−∇·(−PI+1ReT)]ϕkdV,

where** I** is the unit matrix and **T** = ∇**U** + (∇**U**)^*T*^ is the stress tensor of the Newtonian fluid. By applying the divergence theorem in order to decrease the order of differentiation, (4) reduces to

(5) RMk=∫V[(∂U∂t+U·∇U)ϕk+(−PI+1ReT)·∇ϕk]dV−∫Sn·[−PI+1ReT]ϕkdS.

The volume integral in (5) along with (3) is evaluated at all the interior nodes of the computational domains. The inclusion of the surface integral in (5) at all exit nodes of the domains essentially results in the imposition of the free boundary condition. By not imposing any arbitrary boundary condition at the exits of the computational domains the outflow is given by the exact solution of the governing equations minimizing in this way any outflow distortion that may propagate towards the interior of the domains. The nonlinear algebraic equations system resulting from (3) and (5) is solved with the Newton-Raphson iterative scheme. In Table 1 flow domain tessellations are shown in relation to the number of finite elements, nodes, and unknowns.

## 3. Results and Discussion

The results are presented here in the following sequence: (1) instantaneous and mean streamlines, (2) mean velocity fields, (3) turbulent intensities and Reynolds stresses, and (4) turbulent kinetic energy. The statistical analysis of the turbulent flow is based on the standard equation for the calculation of mean variable quantities:

(6) $$\begin{array}{c} \langle X\rangle =\frac{1}{T_{n}}\overset{t}{\underset{0}{\int }}Xdt, \end{array}$$

where, in our case, the instantaneous flow quantities, *X*, are obtained from the direct solution of the incompressible, dimensionless Navier-Stokes and continuity equations.

The streamwise velocity *U*_1_′ and transverse velocity *U*_2_′ fluctuations are computed by decomposing the instantaneous velocities into their mean and fluctuating parts,

(7) $$\begin{array}{c} U_{1}^{\prime }=U_{1}-\langle U_{1}\rangle ,\\ U_{2}^{\prime }=U_{2}-\langle U_{2}\rangle . \end{array}$$

### 3.1. Instantaneous and Mean Streamlines

**Figure 2:**
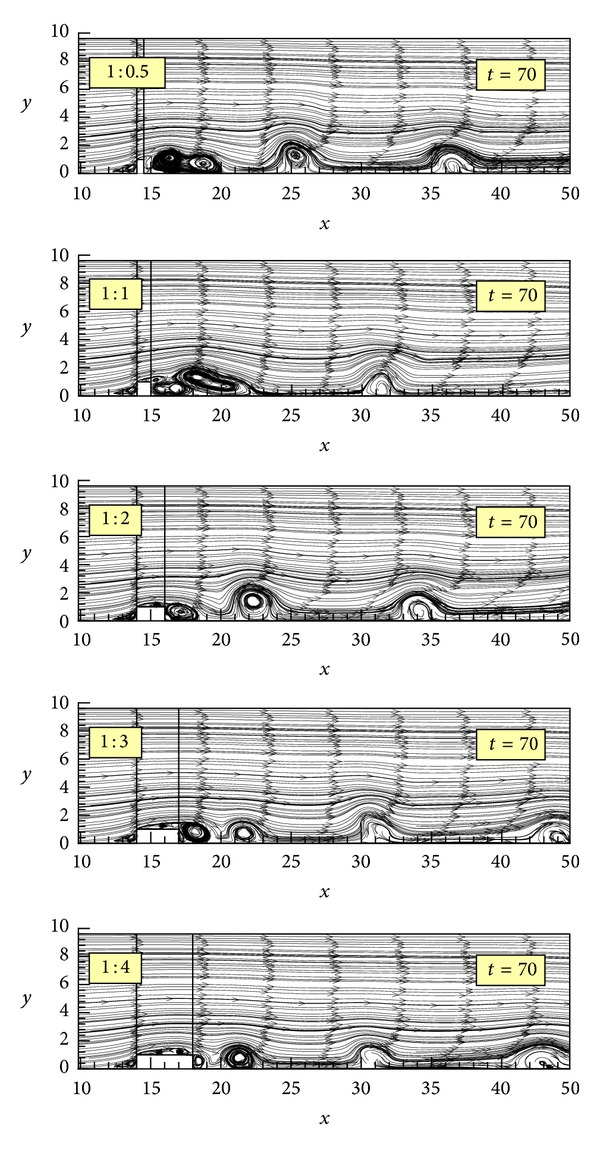
Instantaneous streamline flow patterns for *t* = 70 for the five rib cases.

A time instant of simulated streamlines is shown in Figure 2, for five rib aspect ratios (1 : 0.5, 1 : 1, 1 : 2, 1 : 3, 1 : 4) at *t* = 70. At this particular time instant, the phenomenon of vortex shedding in its full development downstream of the rib is clearly demonstrated. In all cases, the isolated vortical structures are produced at the right corner of the rib and their shape changes in a random way as they travel downstream. Their continuous number and shape variation in the shear layer region lead to a periodical motion of the reattachment point, as has been previously demonstrated by Fragos et al. [21]. Vortices are also produced upstream and over the rib due to the separation of the flow with the over-the-rib vortical structures being more pronounced as the rib aspect ratio increases. A very interesting feature that one notices is the way vortices behave just downstream of the rib. While at small rib ratios, particularly for 1 : 0.5 and 1 : 1 ratios, merged-like vortical structures are observed that are rather difficult to be distinguished, greater rib ratios have the effect of severing the vortical flows resulting in well-formed, individual eddies.

The separation point upstream of the rib appears to be unaffected by the change of the rib length. However, it should be mentioned that the upstream separation point is moving with time, changing the shape of the recirculation region, as it has been previously demonstrated by Fragos et al. [21]. Additionally, there is no apparent relationship between the ribs' length and the vortices' shape; they rather seem to change their shape in a random fashion without following any particular formation pattern.

**Figure 3:**
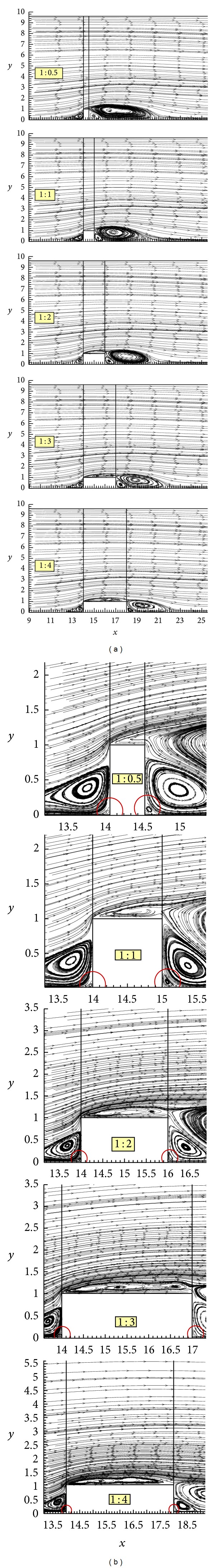
Mean streamline patterns for the five different rib aspect ratios (a) and zoomed-in averaged flow patterns in the neighborhood of the ribs both upstream and downstream (b).

The calculated mean streamlines for the five different rib aspect ratios are shown in Figure 3(a). It can be observed that the shape of the recirculated flow upstream of the rib stays rather stable as rib ratio increases with the only exception of 1 : 2 rib ratio where recirculation flow demonstrates a considerable shrinkage. Concerning the recirculation zone downstream of the rib, it is generally decreasing in both extent and intensity; the longer the rib is the less extensive and less intense the recirculation zone is.

In Figure 3(b) zoomed-in averaged flow profiles for all five aspect ratios are shown. In this figure the capability of our numerical scheme of resolving the tertiary eddies being present in the lower corners of the ribs (thin open red circles) is demonstrated. The capturing of these small-scale vortical flows under time-averaged conditions has an additional value since it implies that these structures are a permanent (at least for the time scale we use) and not a transient feature of the adjacent-to-the-rib flows.

**Figure 4:**
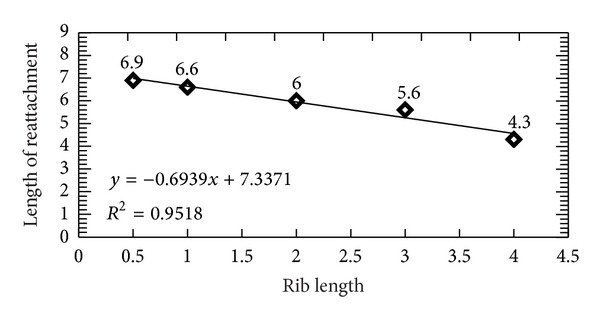
Length of reattachment under different rib lengths.

By closer inspection of Figure 3(a) we were able to define with high accuracy the length and thus the point of reattachment downstream of the ribs. The reattachment point was found by determining the *x*-location where the dividing streamline reattaches on the floor.  Figure 4 shows the linear fitting of the five reattachment points which are defined in relation to the rib lengths. The coefficient of determination, *R* ^2^, is estimated to be 0.9518 which is a relatively high value. This indicates that the linear fitting defined can be used as a prediction tool to estimate the lengths of reattachment under variable rib lengths. This is very useful especially in solar air heater applications where the recirculation region extent is the factor that mainly affects the heat transfer coefficient [11].

**Figure 5:**
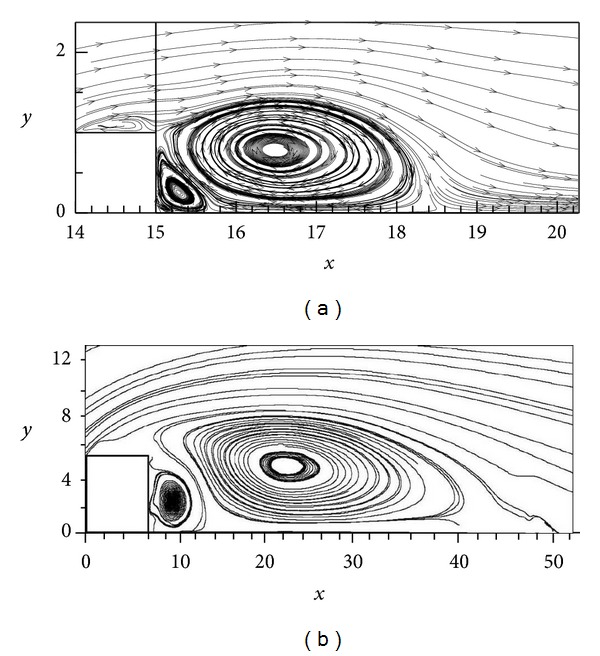
Comparison of streamline patterns under 1 : 1 rib aspect ratio and *Re* = 1920. (a) Results from our numerical method, while (b) is taken from Agelinchaab and Tachie [16].

In order to strengthen the efficiency of our numerical method, in Figure 5, a qualitative comparison is made with the experimental results shown in the upper panel of Figure 3 in Agelinchaab and Tachie [16] for the square rib case. The comparison of the computed flow streamlines of the present study with the streamlines of the above laboratory experiment, for *Re* = 1920, and the sense of vortex rotation, with the downstream primary and secondary eddies demonstrating a counterclockwise and clockwise sense of recirculation, respectively, confirms that the present numerical model is simulating satisfactorily the studied airflow patterns. The difference in the recirculation length of the flow between the simulation and the experiment is due to the different upstream boundary conditions, the thickness of the boundary layer, and the turbulence level. More specifically, Agelinchaab and Tachie [16] are using a turbulence level of 5% which is very close to the turbulence level that is considered in the present study. Therefore the differences in flow conditions are mainly due to the different initial conditions. In both panels, two pairs of separations and reattachments are found with the exception of a small separation bubble evident on top of the rib for the numerical case (Figure 5(a)). Similar direct numerical simulations have been conducted using the authors' code for different obstacles geometries in wind tunnel/duct and the validity of the code and its respective numerical results were verified by laboratory experiments carried out in a wind tunnel [20].

### 3.2. Mean Velocity Fields

**Figure 6:**
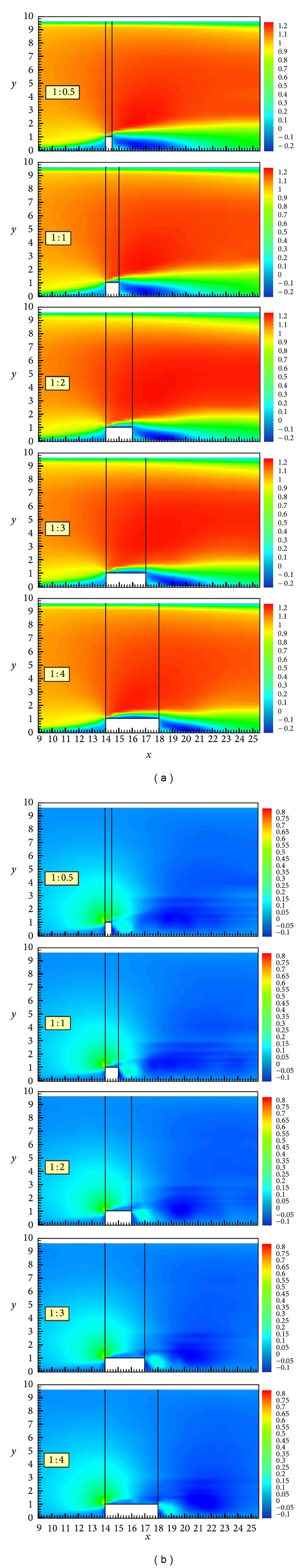
Color-coded plots of streamwise mean velocity 〈*U*_1_〉 (a) and transverse mean velocity 〈*U*_2_〉 (b).

Figures 6(a) and 6(b) show the streamwise and transverse mean velocity color-coded profiles, respectively, for the whole computational domain. As it is evident, the velocity profiles in the recirculation region downstream of the ribs deviate significantly from the upstream profiles, in qualitative agreement with the experimental results of Agelinchaab and Tachie [16]. The streamwise mean flow close to the base is almost equally distorted for all rib aspect ratios, although the dividing streamlines, as defined in Agelinchaab and Tachie [16], have significantly variable points of reattachment relative to the rib lengths, as it is verified by the relative extent of the recirculation zones when looking at Figure 3(a). By close comparison of left and right panels we can note that the streamwise mean velocity 〈*U*_1_〉 is quite comparable to the transverse mean velocity 〈*U*_2_〉 inside the recirculation region downstream of the ribs. This is particularly true at 1 distance unit downstream of the ribs where the velocity ratio is almost 1 providing a numerical confirmation for the experimental results of Agelinchaab and Tachie [16] who have demonstrated that for a square rib the classical thin shear-layer assumption where 〈*U*_2_〉 is small compared to 〈*U*_1_〉 is invalid in the recirculation region close to the base wall.

### 3.3. Turbulent Intensities and Reynolds Stresses

**Figure 7:**
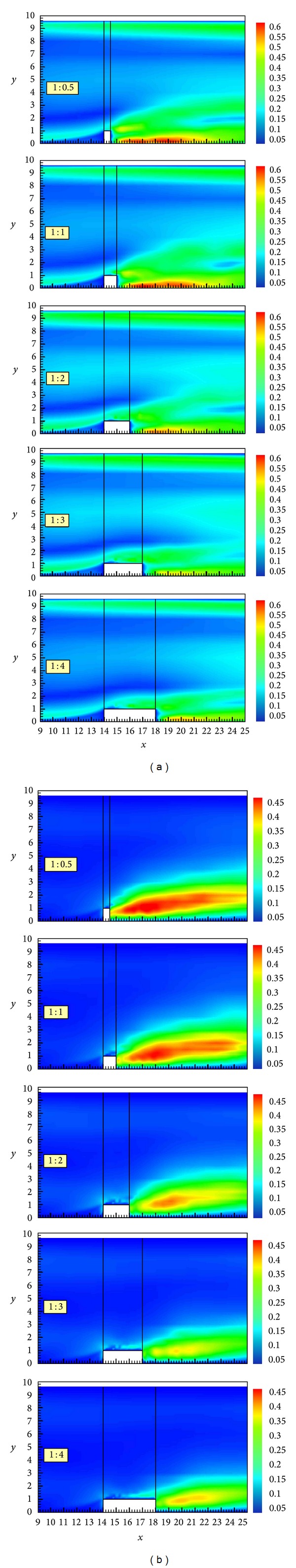
Color-coded plots of streamwise (a) and transverse (b) turbulent intensity.

**Figure 8:**
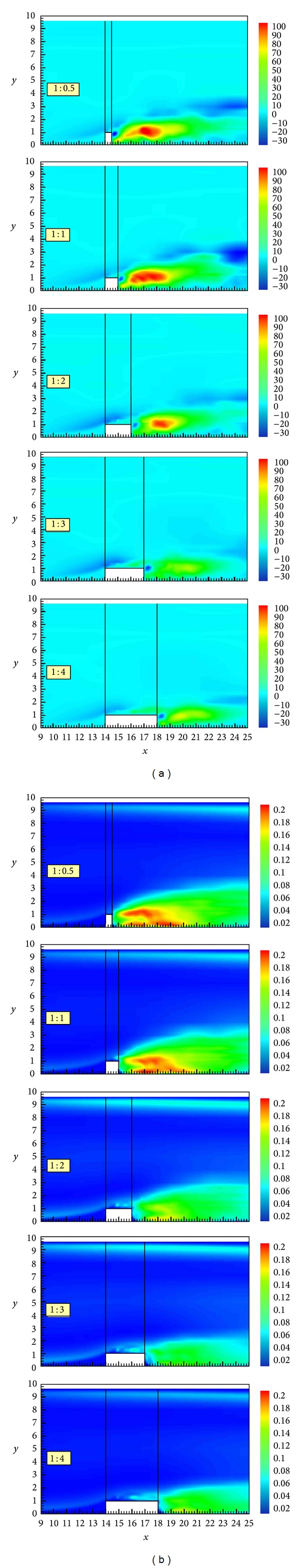
Color-coded plots of Reynolds stresses (a) and turbulent kinetic energy (b).

The magnitudes of streamwise turbulent intensity $\sqrt{\langle {U_{1}^{\prime }}^{2}\rangle }$ and transverse turbulent intensity $\sqrt{\langle {U_{2}^{\prime }}^{2}\rangle }$ for the five different rib lengths are shown in Figure 7, while Reynolds shear stress −〈*U*_1_′*U*_2_′〉 is shown in Figure 8(a). Streamwise turbulent intensity maximizes downstream of the ribs in the recirculation region close to the base wall, while its maximum value diminishes as we proceed to higher rib lengths. Similar to the streamwise, transverse turbulent intensity also peaks downstream of the ribs; however, the extent of the maximum values is much higher in both streamwise and transverse directions. It is also worth noting that the region of peak values tends upwards away from the base wall, while their magnitudes gradually diminish as we proceed to greater rib lengths, similar to the streamwise case.

The computed values of Reynolds stresses (Figure 8(a)) maximize downstream of the ribs and by reexamining Figure 3(a) we do observe that the regions of maximization coincide with the cores of the primary flow eddies. This is something expectable since Reynolds stress represents the average momentum flux due to the velocity fluctuations, characterizing the transfer of momentum by turbulence [22].

### 3.4. Turbulent Kinetic Energy

The turbulent kinetic energy (TKE) is given by the equation

(8) $$\begin{array}{c} k=\frac{1}{2}\left(\langle {U_{1}^{\prime }}^{2}\rangle +\langle {U_{2}^{\prime }}^{2}\rangle \right), \end{array}$$

which is characterized by the measured root-mean-square (RMS) velocity fluctuations, that is, the turbulent intensities already plotted in Figure 7. Hence, as it is evident in Figure 8(b), turbulent kinetic energy can be considered as the superposition of the corresponding panels in Figure 7. By close inspection we do see that TKE follows the same dissipation pattern as we proceed to higher rib lengths. Concerning the production of TKE, −〈*U*_*i*_′*U*_*j*_′〉(∂〈*U*_*i*_〉/∂〈*ω*_*j*_〉), the discrepancy between Reynolds stresses and turbulent kinetic energy is apparently attributed to the fact that the production of TKE is also affected by the strain rate (gradient of mean velocity) which in the case of incompressible flows is linearly dependent on viscous stress.

## 4. Conclusions

The present paper investigates the two-dimensional flow around a rib with five different aspect ratios using the Direct Numerical Simulation method. The study concentrates on the characteristics of the fluid flow passing through a duct simulating a solar air heater with single rectangular ribs as roughness elements being attached on one wall. The analysis clearly shows how the rib length can affect the extent of the recirculation region and thus the point of reattachment downstream of the rib. The extent and intensity of turbulence are more pronounced for smaller rib lengths, something which is confirmed by both the lengths of reattachment and the turbulent intensity profiles. This fact clearly suggests that as we proceed to smaller rib element lengths a simultaneous increase in heat transfer efficiency will occur.

The analysis although carried out for single rectangular ribs of different aspect ratios can be extended to include cases of multiple ribs of different geometries, for example, pitched- or arched-shaped ribs. Such an analysis can contribute significantly to the investigation of how the pitch ratio and roughness element geometries would affect the flow pattern and consequently the heat transfer coefficient.

## Supplementary Material

6 video clips demonstrating streamline patterns evolution, streamwise and transverse instantaneous velocity evolution for two different rib aspect ratios and Reynolds number 1000.

## Figures and Tables

**Table 1:** Data of the computational meshes shown in Figure 1.

Rib aspect ratio	Number of elements	Number of nodes	Number of unknowns
1 : 0.5	14995	60699	136753
1 : 1	14645	59299	133603
1 : 2	14970	60629	136603
1 : 3	15350	62181	140103
1 : 4	14750	59781	134703
